# Covalent Organic Framework Nanofilm Heterojunctions: Lamination Effect and Suppressed Self‐Discharge in Flexible Micro‐Supercapacitors Energy Storage

**DOI:** 10.1002/smll.202412642

**Published:** 2025-03-27

**Authors:** Xiaoyang Xu, Tian Li, Ruijuan Zhang, Zihao Zhang, Wei Cao, Yue Wang, Yongqi Hu, Xinying Liu, Shanlin Qiao

**Affiliations:** ^1^ College of Chemistry and Pharmaceutical Engineering Hebei University of Science and Technology Shijiazhuang 050018 China; ^2^ Institute for Catalysis and Energy Solutions University of South Africa Private Bag X6, Florida Roodepoort 1710 South Africa

**Keywords:** covalent organic framework nanofilms, energy storage, micro‐supercapacitors, self‐discharge

## Abstract

Covalent organic frameworks (COFs) nanofilms with well‐ordered channels and highly active interfaces have great potential in in‐plane micro‐supercapacitors (MSCs). COF heterojunction nanofilms integrate the benefits of individual COF phases through alternating stacking. Herein, sandwich‐type COF heterojunctions are prepared under *van der Waals* bonding, controlling the larger outer aperture (vs. inner) to create the stereoscopic lamination effect in axial channel structure, which enhances the rapid transport of electrolyte H⁺ and their concentrated accumulation on active interfaces. Simultaneously, the unique heterojunction structure effectively reduces resistance to electron transport, enabling electrons to conduct through in‐plane π‐electron clouds and facilitating *π–π* electron transitions across interfaces. In addition, the outer aperture in COF heterojunctions is also adjusted to inhibit H^+^ overflow causing the self‐discharge phenomenon. The results show that the optimal MSC‐COF*_1.0‐0.6‐1.0_* exhibits a high volumetric specific capacitance (*C_V_*) of 598.6 F cm^−3^, high energy density of 40.7 mWh cm^−3^ at 2095.2 mW cm^−3^, good self‐discharge property up to 36 h, and excellent cycling and bending‐resistant stability. This work about the optimal integration of multiple factors in COF heterojunctions, including lamination effect, interface contribution, and ion overflow, can provide theoretical guidance for the application of COF heterojunctions in miniature or flexible/wearable devices.

## Graphical abstract

Tailored sandwich‐type COF heterojunctions achieve optimal capacitance and energy density through H⁺ ion lamination and π‐electron conduction, demonstrating the potential for miniaturized and flexible energy storage devices.

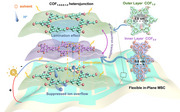

## 1 Introduction

Planar micro‐supercapacitors (MSCs) have emerged as a promising alternative to meet the demands of thinner and more flexible portable electronic devices, offering shorter ion transport distances and enhanced power density, while the challenge remains in achieving a high energy density.^[^ ^1^ ^]^ Compared to thin‐film electrode materials such as graphene, MXene, and metal–organic frameworks,^[^ ^2^, ^3^, ^4^ ^]^ Covalent organic frameworks (COFs) possess unique advantages such as permanent porosity, well‐defined pore sizes, ordered channel structures, and large surface areas.^[^ ^5^, ^6^, ^7^, ^8^ ^]^ By meticulously designing COFs’ structure, electroactive functionalization (COF‐366‐Co),^[^ ^9^ ^]^ and constructing microstructures (nanowires, spheres),^[^ ^10^, ^11^ ^]^ the energy and power density of MSCs can be significantly enhanced. However, the poor processability of COF microcrystalline powders limits their application in MSCs. Therefore, improving the manufacturing technology of COFs films and reducing interface barriers are key research directions for enhancing the performance of MSCs.

In recent years, free‐standing COFs nanofilms, formed through the self‐assembly permutation polymerization,^[^ ^12^ ^]^ offer a robust platform for direct use as thin‐film electrodes with 1D axial ion transport channel. Some worthwhile attempts at these self‐supported nanofilms for MSCs confirm the outstanding superiority of bulk‐to‐film COFs, such as space‐partitioning and metal coordination in free‐standing imine‐linked COF nanofilms: over 230 mWh cm^−3^ energy density for flexible MSCs.^[^ ^13^ ^]^ Furthermore, superlattice configuration assembled alternately by different nanofilms based on *van der Waals* force can integrate the advantages of each isolated layer, and thereby COF superlattice heterostructure was first assembled and applied to bending‐resistance MSCs electrode.^[^ ^14^ ^]^ This indicates that the effective integration of imine‐linked COFs to regulate the inherent porous structure of heterogeneous COF phases can significantly enhance capacitance performance by suppressing self‐discharge. Moreover, the *van der Waals* 2D heterostructure inherits the advantages of individual COF layers and further mitigates self‐discharge through controlled pore arrangement and interfacial charge separation.

The assembly mechanism of this 2D COF superlattice heterostructure relies on non‐covalent *van der Waals* interactions. *Van der Waals* forces refer to the weak interaction between molecules or atoms, including orientation force, induction force, and dispersion force. These forces arise from electrostatic and transient dipolar interactions between molecules.^[^ ^15^ ^]^ Although individual *van der Waals* interactions are weak, their cumulative effects are sufficient to drive spontaneous stacking between different COF phases in the multilayer structure, resulting in stable and well‐integrated heterojunction structures. This weak interaction can effectively avoid the lattice mismatch problem that may be caused by traditional covalent bonding, and achieves efficient electron transport along the plane and charge separation across the interface by promoting *π–π* overlap between layers.^[^ ^16^ ^]^

In this work, a series of COF nanofilms with different aperture structures were customized and prepared by interfacial polymerization, followed by being assembled into sandwich‐type COF heterojunctions. These COF heterojunctions were predesigned in accordance with first adjusting the internal phase aperture at the largest outer aperture of 3.9 nm, and then further regulating the outer phase aperture at the afore‐selected internal aperture. This designed concept is as follows: i) the largest outer aperture of 3.9 nm is for obtaining the high‐concentration electrolyte ion accumulation in the inner COF layer of heterojunctions during the charging process; ii) the outer phase aperture at the afore‐selected internal aperture was further regulated, for minimizing self‐discharge ability from the electrolyte ion overflow in the outer large aperture. The obtained COF*_x‐y‐x_* (*x, y* represent outer, and internal phase aperture, respectively) heterojunctions were directly established into flexible MSCs, which were systematically characterized in terms of chemical structure and electrochemical performances. The results show that the COF*_1.0‐0.6‐1.0_* heterojunction exhibits remarkably high activity as flexible electrodes for in‐plane MSC.

## 2 Results and Discussion

### 2.1 Sample Characterization

**Scheme 1:**
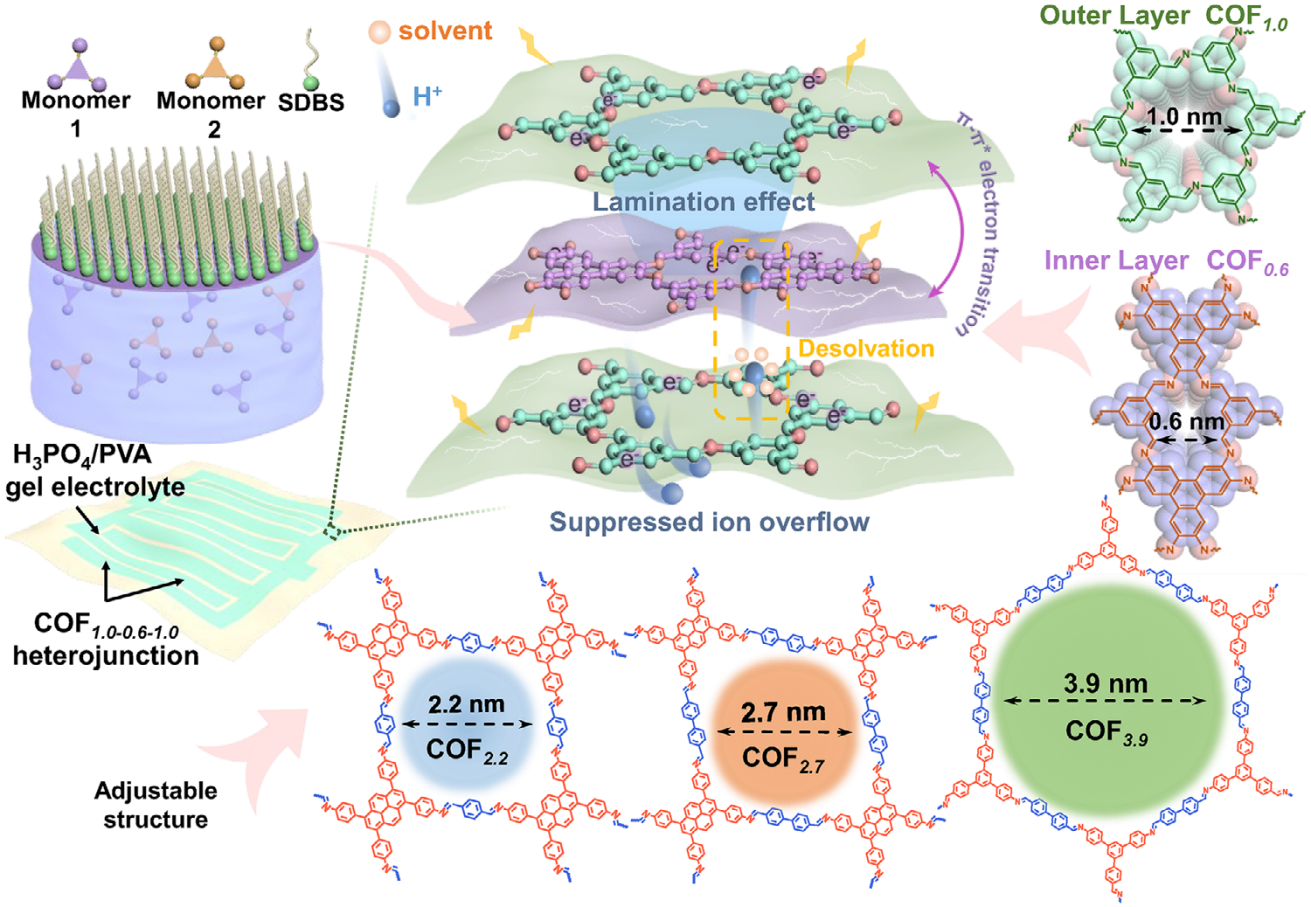
Molecular design and synthetic engineering of COF nanofilms and heterojunctions for MSC application.

The COFs molecular design was performed mainly by taking the theoretical aperture structure in Figure S1a–e (Supporting Information) as a reference, such as COF*_0.6_* from the imine condensation reaction of HATP and TFB, COF*_1.0_* from BTCA and TFB, COF*_2.2_* from PyTA and PDA, COF*_2.7_* from PyTA and BDC, COF*_3.9_* with TAPB and BDC monomers. As shown in **Scheme** **1**, under the orientation guidelines of surfactants, these monomers are successively diffused to the gas‐liquid interface in order to polymerize, in which the formed prepolymer is orderly self‐assembled to obtain the free‐standing COF*_n_* nanofilms. Then these COF*_n_* nanofilms were recombined in a predesigned order under *van der Waals* bonding, to form sandwich‐type COF*_x‐y‐x_* heterojunctions, as COF*_1.0‐0.6‐1.0_* with COF*_1.0_* outer layer and COF*_0.6_* inner layer for example. The COF heterojunctions interdigital electrode was well‐connected with PVA/H_3_PO_4_ gel electrolyte and assembled into a MSC device. The stereochemical structure of COF*_x‐y‐x_* heterojunctions was further optimized by energy storage and self‐discharge performance.

These COF*_n_* (*n* = 0.6, 1.0, 2.2, 2.7, 3.9) nanofilms were characterized by Fourier transform infrared spectrometer (FT‐IR) and X‐ray photoelectron spectroscopy (XPS) with respect to chemical composition and skeleton structure. In FT‐IR spectra of Figure S1f (Supporting Information), an obvious characteristic peak at≈1630 cm^−1^ occurs in these COF films, corresponding to tensile vibration resulting from the condensation reaction between the ─NH_2_ and ─CHO groups, which is strong evidence of the intermonomer connection of imine‐COF.^[^ ^17^ ^]^ Further, the nearly vanished N─H stretching absorption peaks of amino groups in the initial organic monomers also can confirm the conversion of monomers into imine‐COF. The detailed structure of these COF nanofilms can be determined by XPS characterization as follows: the C, N, and O elements can be recognized as expected in molecular chemical composition analysis (Figure S1g, Supporting Information); In XPS C1*s* (Figure S1h, Supporting Information) spectra, the deconvolution peaks at≈284.9 and 285.5 eV are C─C/C═C and imine C ═N,^[^ ^18^ ^]^ respectively; Similarly, these N 1*s* spectra (Figure S1i, Supporting Information) are divided into C─N at 399.7 eV and C═N at 398.5 eV.^[^ ^19^ ^]^ This once again provides strong evidence for the successful synthesis of COFs.

In the FT‐IR spectra of bulk COFs, the imine bond shows stretching vibration peaks in the range of 1620–1670 cm^−1^ and bending vibration peaks in the range of 1250–1270 cm^−1^, respectively (Figure S2a–e, Supporting Information), further verifying the formation of COFs. Furthermore, Figure S3a–e (Supporting Information) shows the powder X‐ray diffraction patterns of bulk COF*_n_* (*n* = 0.6, 1.0, 2.2, 2.7, 3.9), which can be used to reflect the Bulk COF*_n_* (*n* = 0.6, 1.0, 2.2, 2.7, 3.9) nanofilms. The diffraction peak of (100) crystal plane appears at 2θ = 6.88° for COF*_0.6_*, 2θ = 3.40° for COF*_1.0_*, 2θ = 3.34° for COF*_2.2_*, 2θ = 3.24° for COF*_2.7_* and 2θ = 2.26° for COF*_3.9_*, which can further calculate the pore size of COF*_0.6_* ≈0.6 nm, COF*_1.0_* ≈1.0 nm, COF*_2.2_* ≈2.2 nm, COF*_2.7_* ≈2.7 nm and COF*_3.9_* ≈3.9 nm. As shown in Figure S4a–c (Supporting Information), using COF*_1.0‐0.6‐1.0_* as a representative example, we tested the *Young's* modulus of the heterojunction and their monolayers, which is a key point to the strength of nanofilms. *Stress tolerance* tests of COF*_0.6_*, COF*_1.0,_* and COF*_1.0‐0.6‐1.0_* films show that their elastic modulus can reach 2.8 GPa for COF*_0.6_*, 2.6 GPa for COF*_1.0_* and 10.7 GPa for COF*_1.0‐0.6‐1.0_*, respectively, which proves their good mechanical properties.

**Figure 1:**
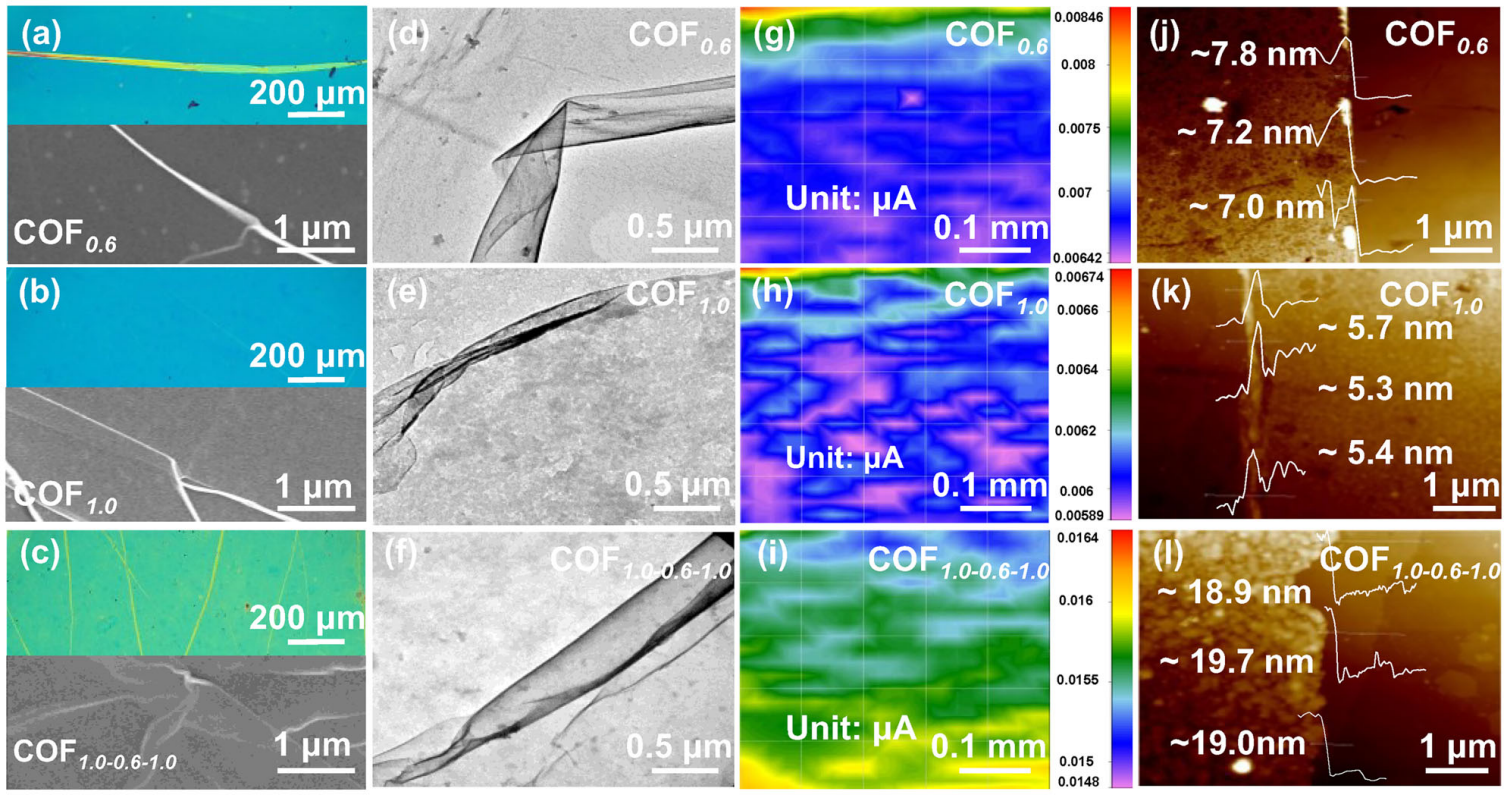
a–c) Images of OM and SEM, d–f) TEM, g–i) SECM, j–l) AFM images of COF*_0.6_*, COF*_1.0_*, and COF*_1.0‐0.6‐1.0_*.

Taking COF*_1.0‐0.6‐1.0_* heterojunctions as a representative, including COF*_0.6_* and COF*_1.0_* films, were characterized using optical microscopy (OM), scanning electron microscopy (SEM), transmission electron microscopy (TEM), and scanning electrochemical microscopy (SECM). In **Figure** **1**a–f, the synthesized COF*_0.6_*, COF*_1.0_* nanofilms, as well as COF*_1.0‐0.6‐1.0_* heterojunction, are all smooth and uniform large‐area films. The monitoring maximum current difference of Fe^2+^/Fe^3+^ redox in the 0.5 × 0.5 mm scanning region is ≈0.97 nA in 2D SECM images (Figure 1g–i), further confirming the flat and intact surface structure in large‐area scale of COF*_0.6_*, COF*_1.0_* nanofilms, and COF*_1.0‐0.6‐1.0_* heterojunction. These results demonstrate the nondestructive transfer by layer‐by‐layer and well‐connected recombination for these nanofilms. Notably, these COF films can be transferred into other substrates without destroying the integrity of films, also confirming the excellent mechanical strength. After atomic force microscopy (AFM) test (Figure 1j–l), the thickness of COF*_1.0‐0.6‐1.0_* heterojunction was measured to be ≈19.2 nm, along with 5.5 and 7.3 nm for COF*_1.0_*, and COF*_0.6_* nanofilms. The measured thickness of COF*_1.0‐0.6‐1.0_* heterojunctions (≈19.2 nm) is slightly larger than the sum of 2COF*_1.0_* + COF*_0.6_* (≈18.5 nm), which may be due to *van der Waals* forces between the layers and surface absorption of water or organic molecules.^[^ ^20^ ^]^

### 2.2 Investigation of Charge Storage Capacity and Mechanism

**Figure 2:**
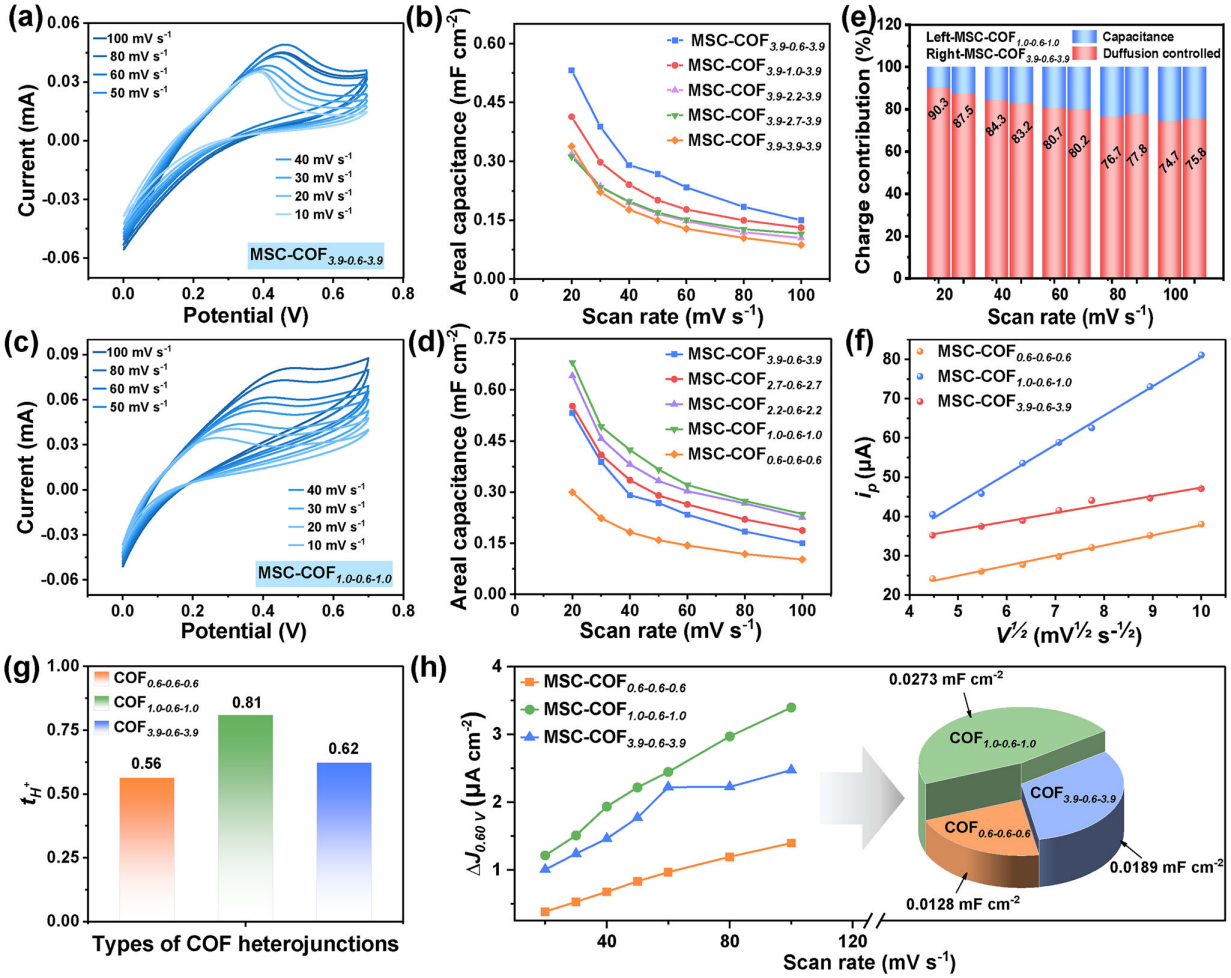
a) *C–V* curves and b) *C_A_* of MSC‐COF*_3.9‐y‐3.9_* (*y* = 0.6, 1.0, 2.2, 2.7, 3.9); c) *C–V* curves and d) *C_A_* of MSC‐COF*_x‐0.6‐x_* (*x* = 0.6, 1.0, 2.2, 2.7, 3.9); e) diffusion‐controlled and surface capacitance contribution of MSC‐COF*_1.0‐0.6‐1.0_*, and MSC‐COF*_3.9‐0.6‐3.9_*; f) plots of *i_p_* versus *v^1/2^* for MSC‐COF*_0.6_*_‐_ *_0.6_*_‐_ *_0.6_*, MSC‐COF*_1.0_*_‐_ *_0.6‐1.0_*, and MSC‐COF*_3.9‐0.6‐3.9_*; g) $t_{H^{+}}$ of COF*_1.0‐0.6‐1.0_*, and COF*_3.9‐0.6‐3.9_* heterojunctions; h) Capacitive current density of MSC‐COF*_0.6‐0.6‐0.6_*, MSC‐COF*_1.0‐0.6‐1.0_*, and MSC‐COF*_3.9‐0.6‐3.9_* heterojunctions at 0.60 V as a function of scan rates, direct comparison of *C_dl_* values in pie chart.

After the interdigital treatment, the COF*_x‐y‐x_* heterojunctions were assembled into in‐plane MSCs, defined as MSC‐COF*_x‐y‐x_*, which can be characterized by cyclic voltammetry (CV) measurement in electrochemical properties. First, a 3.9 nm COF framework is used as the outermost layer to ensure the optimal interlayer effect is achieved. The energy storage capacities of series MSC‐COF*_3.9‐y‐3.9_* (*y* = 0.6, 1.0, 2.2, 2.7, 3.9) devices were evaluated by *C–V* curves, as shown in **Figure** **2**a; Figure S5a–d (Supporting Information), some visible peaks come from a redox reaction where nitrogen in C═N bonds lose an electron, turning into a positively charged imine cation.^[^ ^21^ ^]^

By comparing the *C–V* curves at the same scanning rate, it was found that among the COF*_3.9‐y‐3.9_* heterojunctions, MSC‐COF*_3.9‐0.6‐3.9_* exhibits the largest response current and closed integral area, which is directly related to its maximum specific capacitance. According to the calculation results from Equation S1 (Supporting Information), the calculated areal specific capacitance (*C_A_*) of MSC‐COF*_3.9‐0.6‐3.9_* is significantly better than that of other COF*_3.9‐y‐3.9_* heterojunctions (Figure 2b). This is because electrolyte ions can diffuse through the external large‐pore channels and moderately aggregate on the 0.6 nm internal pore interface with abundant active sites, thereby enhancing its energy storage capacity.^[^ ^22^ ^]^ As the inner aperture (*y*) gradually increases, the *C*_*A*_ of the COF_*3.9‐y‐3.9*_ heterojunctions gradually decreases, which can be attributed to the weakening of the gradient pore diameter's layering effect in the 3D structure.

Considering the non‐negligible decreasing active interface for the outer large‐aperture channel, Figure 2c and Figure S6a–d display the *C–V* curves of the tunable MSC‐COF*_x‐0.6‐x_* devices (*x* = 0.6, 1.0, 2.2, 2.7, 3.9) with an internally optimized pore size of 0.6 nm. The CV shape of MSC‐COF*_1.0‐0.6‐1.0_* in Figure 2c is similar to that of MSC‐COF*_3.9‐0.6‐3.9_*, which is attributed to the analogous COF frameworks of the heterojunctions. At scan rates of 20, 30, 40, 50, 60, 80, and 100 mV s^−1^, MSC‐COF*_1.0‐0.6‐1.0_* exhibits superior specific capacitance values within the MSC‐COF*_x‐0.6‐x_* series (Figure 2d), with specific values of 0.24, 0.27, 0.32, 0.37, 0.42, 0.49, and 0.68 mF cm^−2^. In Figure S7a (Supporting Information), the areal‐specific capacitance value of MSC‐COF*_1.0‐0.6‐1.0_* is significantly higher than that of MSC‐COF*_1.0_* and MSC‐COF*_0.6_* at the scan rates of 20, 30, 40, 50, 60, 80, and 100 mV s^−1^. It is further confirmed that the lamination effect is beneficial for energy storage. Consequently, after optimizing the internal and external aperture of stereoscopic heterojunctions, COF*_1.0‐0.6‐1.0_* demonstrates the highest capacitive performance among the COF*_x‐y‐x_* heterojunctions, achieving an optimal balance between the active interface and lamination effects.

Figure 2e illustrates the charge storage mechanisms of MSC‐COF*_1.0‐0.6‐1.0_* and MSC‐COF*_3.9‐0.6‐3.9_*, which include the diffusion‐controlled and surface capacitive contributions, as analyzed based on equation (S5, Supporting Information). The diffusion dominates in the overall charge storage process of these two devices, even though the diffusion‐controlled contribution rate decreases from 90.3% to 74.7% for MSC‐COF*_1.0_*_‐_ *_0.6_*_‐_ *_1.0_* at the increasing scan rates from 20 to 80 mV s^−1^, and from 87.5% to 75.8% for MSC‐COF*_3.9_*_‐_ *_0.6_*_‐_ *_3.9_*. This diffusion‐dominated charge storage is particularly evident in the pseudo‐capacitance of MSCs, bringing in faradaic redox reaction at the lattices or near‐surface active sites, except for the double electric layer at the electrode/electrolyte interface.^[^ ^23^ ^]^ At low scan rates of 20–60 mV s^−1^, the diffusion‐controlled ratio of MSC‐COF*_1.0_*_‐_ *_0.6_*_‐_ *_1.0_* is higher than that of MSC‐COF*_3.9_*_‐_ *_0.6_*_‐_ *_3.9_*, while instead lower at high scan rates of 80–100 mV s^−1^. The reason is as follows: The COF*_1.0‐0.6‐1.0_* heterojunction has ample response time at low scan rates, allowing for sufficient redox reactions to occur at the abundant small‐pore interfaces; while the COF*_3.9_*_‐_ *_0.6_*_‐_ *_3.9_* heterojunction with large aperture can achieve rapid diffusion of electrolyte ions at high scan rates.^[^ ^24^ ^]^ This diffusion‐controlled charge storage mechanism can be also demonstrated by the peak current *i_p_* versus *v* ^1/2^ plots. As shown in Figure 2f, the straight line of *i_p_* versus *v* ^1/2^ plots is on behalf of b≈0.5 for MSC‐COF*_0.6_*_‐_ *_0.6_*_‐_ *_0.6_*, MSC‐COF*_1.0_*_‐_ *_0.6‐1.0_* and MSC‐COF*_3.9‐0.6‐3.9_*, further indicating the aforementioned faradaic redox behavior during charging and discharging.^[^ ^25^ ^]^

As shown in Figure 2g, the H⁺ transference numbers for the electrolytes in the COF*_1.0‐0.6‐1.0_*, and COF*_3.9‐0.6‐3.9_* heterojunctions are 0.81 and 0.62, respectively, both of which exceed the 0.56 of COF*_0.6‐0.6‐0.6_*. The dynamic EIS spectra (Figure S7b, Supporting Information) of these nanofilms exhibit negligible semi‐circular arcs and straight lines in the high and low‐frequency regions, which correspond to the low charge‐transfer and *Warburg* resistances. This result highlights the significant role of the stereoscopic lamination effect in accelerating the migration rate of H⁺ ions. However, the H^+^ storage rate at inner COF*_0.6_* layers seriously lags behind the migration rate across outer COF*_3.9_* layers, resulting in multiple migrations, leading to the lower H^+^ transference number of COF*_3.9‐0.6‐3.9_* than COF*_1.0‐0.6‐1.0_* heterojunctions. The mentioned H^+^ storage rate is determined by the accessible active interface, which can be quantified by the double‐layer capacitance (*C_dl_*) value.^[^ ^26^ ^]^ Figure 2h shows the relationship of current density difference (Δ*J*) with scan rates for different MSC‐COF*_x‐y‐x_* at 0.60 V. Based on these data, the calculated *C_dl_* values of MSC‐COF*_0.6‐0.6‐0.6_*, MSC‐COF*_1.0‐0.6‐1.0_*, and MSC‐COF*_3.9‐0.6‐3.9_* are 0.0128, 0.0273, and 0.0189 mF cm^−2^, respectively. The pie chart intuitively shows that COF*_1.0‐0.6‐1.0_* surpasses COF*_0.6‐0.6‐0.6_*, and COF*_3.9‐0.6‐3.9_* in an effective active interface. These results strongly demonstrate that the charge storage capacity of COF*_x‐y‐x_* heterojunctions depends on the lamination effect derived from the 3D porous channels and the synergistic contribution of the effective active interfaces.

**Figure 3:**
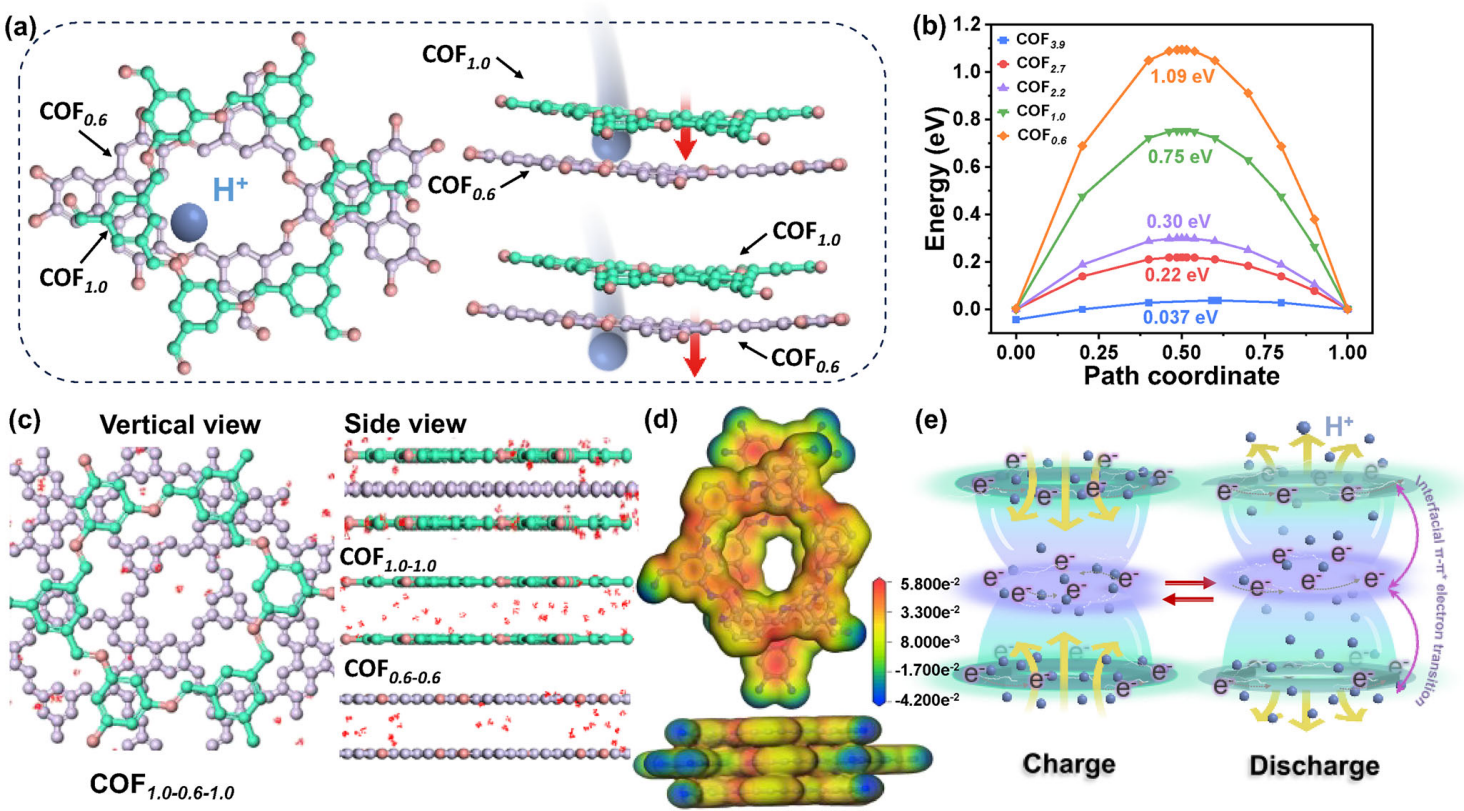
a) Proton diffusion paths across a vertical direction through COF*_0.6_* and COF*_1.0_* adjacent phases in representative COF*_1.0‐0.6‐1.0_* heterojunction; b) Proton diffusion barriers of COF*_n_* phases; c) Proton adsorption simulation diagram of COF*_1.0‐0.6‐1.0_* heterojunction, COF*_1.0‐1.0_* and COF*_0.6‐0.6_* structure; d) Electrostatic potential distribution view of COF*_1.0‐0.6‐1.0_* heterojunction (yellow‐red area indicates charge accumulation, cyan‐blue area indicates charge depletion); e) Electrolyte ions/electrons migration of heterojunctions during charge and discharge process.

The active sites and H^+^ diffusion kinetics in heterojunctions were further verified by time‐dependent density functional theory calculation. **Figure** **3**a provides the proton diffusion paths in the representative COF*_1.0‐0.6‐1.0_* heterojunction, including the proton traveling in a vertical direction through COF*_0.6_*, and COF*_1.0_* adjacent phases. In this regard, proton diffusion barriers of COF*_n_* phases were theoretically calculated by the electrostatic potential diagram (Figure 3b). As the aperture increases from COF*_0.6_* to COF*_1.0_*, the proton diffusion barrier decreases from 1.09 to 0.75 eV, indicating that COF*_1.0_* is more conducive to the diffusion process of protons compared to COF*_0.6_*. As shown in Figure 3c, the COF*_1.0‐0.6‐1.0_* heterojunction exhibits a higher proton adsorption density than COF*_1.0‐1.0_* and COF*_0.6‐0.6_*, in which protons can achieve fast‐diffusion through outer COF*_1.0_*, and accumulated/stored in inner COF*_0.6_* phase, resulting in a synergic effect of protons diffusion and storage. In the electrostatic potential distribution map of Figure 3d, the junction of COF*_1.0_* and COF*_0.6_* exhibits regions of charge accumulation and depletion (yellow‐red represents charge accumulation, while cyan‐blue represents charge depletion). Studies show that the active sites with high electron cloud density can enhance the redox activity for high pseudo‐capacitance.^[^ ^27^ ^]^ Especially in the interfacial proximity areas of the COF*_1.0‐0.6‐1.0_* heterojunction, a higher charge density is exhibited, which represents a higher electrochemical activity.

In summary, Figure 3e depicts the above‐proven electrolyte ions/electrons migration of heterojunctions during the charge and discharge process: (i) the electrolyte H^+^ ions can rapidly pass through the outer COF*_1.0_* channel with a low proton diffusion barrier. Under the action of the aperture‐derived lamination effect, they accumulate on the abundant active sites at the internal COF*_0.6_* interface. This process not only achieves a high‐concentration electric double layer but also enhances the redox capacitance; (ii) the unique heterojunction structure effectively reduces resistance to electron transport, enabling electrons to conduct through in‐plane π‐electron clouds, while also facilitating *π–π* electron transitions across interfaces, thereby achieving higher power output; (iii) the sandwich‐type heterojunction can avoid the strain and energy storage degradation of inner COF*_0.6_* during repeated charge–discharge cycles, in the case that inner micro‐pores cannot fully withstand the high‐loading H^+^ ions from outer large aperture lamination effect; (iv) the outer COF*_1.0_* can also restrict H^+^ ions overflow causing self‐discharge properties, apart from aperture‐derived lamination effect on inner COF*_0.6_*, as well as charge accumulation layer.

### 2.3 Flexibility and Application of the Fabricated MSC Device

**Figure 4:**
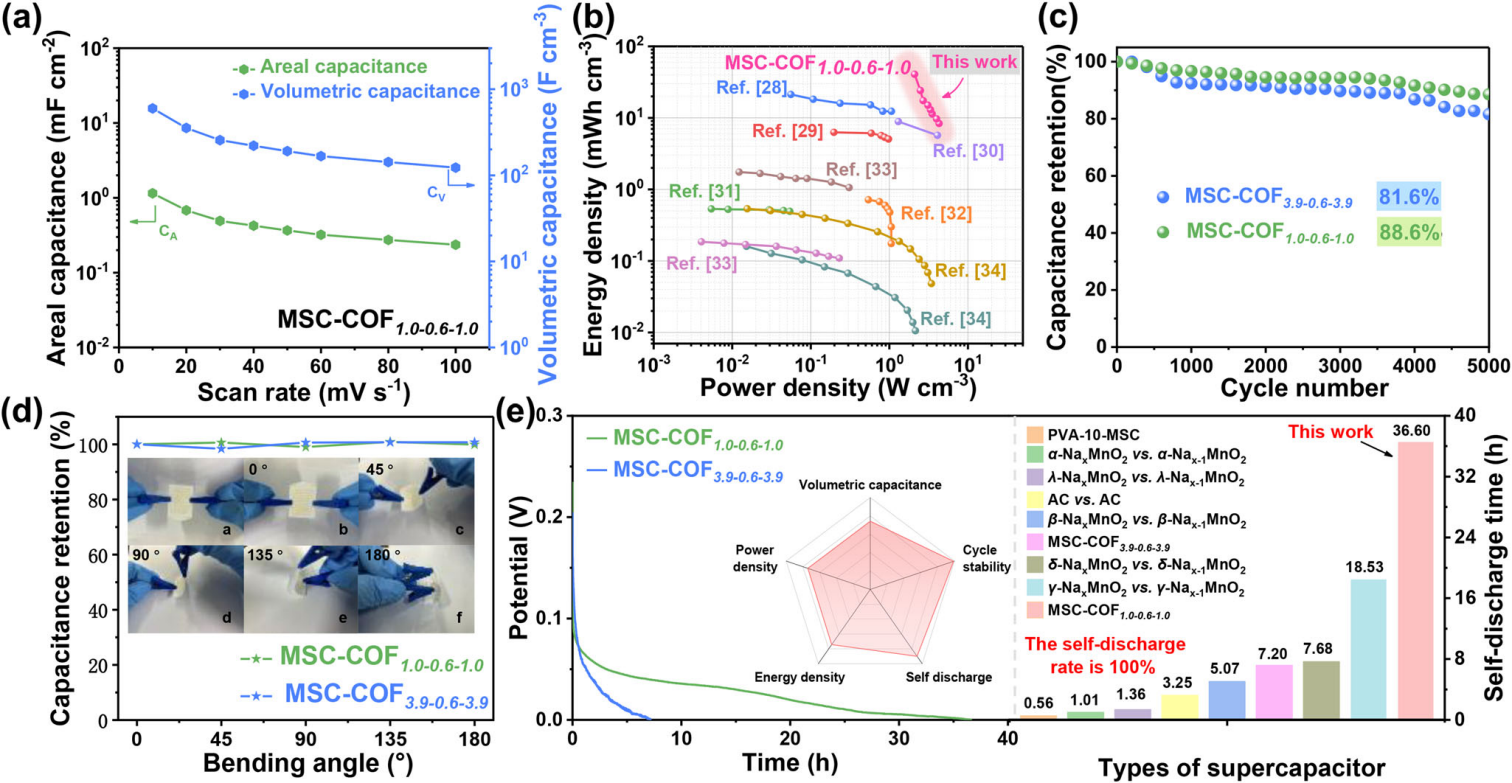
a) *C_A_* and *C_V_* of MSC‐COF*_1.0‐0.6‐1.0_* at different scan rates; b) Ragone plots of our MSC‐COF*_1.0‐0.6‐1.0_* versus reported MSC devices; Capacitance retention c) after 5000 cycles and d) under different bending degrees of MSC‐COF*_1.0‐0.6‐1.0_* and MSC‐COF*_3.9‐0.6‐3.9_*; e) Self‐discharge time of MSC‐COF*_1.0‐0.6‐1.0_* and MSC‐COF*_3.9‐0.6‐3.9_*, comparison with reported works, inset radar map is performance evaluation of MSC‐COF*_1.0‐0.6‐1.0_*.

The optimal MSC‐COF*_1.0‐0.6‐1.0_* was further carried out in electrochemical properties. In **Figure** **4**a, the *C_V_* of MSC‐COF*_1.0‐0.6‐1.0,_* calculated according to equation (S2, Supporting Information), are 122.8, 142.6, 167.2, 190.8, 220.8, 256.4, 354.6, 598.6 F cm^−3^ at scan rates of 10, 20, 30, 40, 50, 60, 80, 100 mV s^−1^, based on the heterojunction thickness of 19.2 nm. The important parameters of energy and power density were calculated according to Equations (S3) and (S4) (Supporting Information) in Figure 4b, coupled with some reported MSC devices. The energy density of MSC‐COF*_1.0‐0.6‐1.0_* can reach 40.7 mWh cm^−3^ at a power density of 2095.2 mW cm^−3^. This performance is superior to reported MSCs, such as α‐Co(OH)_2_‐RGO‐MSC,^[^ ^28^ ^]^ MXene‐based in‐plane solid‐state symmetric MSC,^[^ ^29^ ^]^ g‐C_30_N_6_‐COF,^[^ ^30^ ^]^ printed electrochemically exfoliated graphene MSC,^[^ ^31^ ^]^ commercial activated carbon AC‐SC,^[^ ^32^ ^]^ in‐series screen‐printed graphene MSC,^[^ ^33^ ^]^ and flexible boron‐doped laser‐induced graphene MSC.^[^ ^34^ ^]^

To evaluate the long‐term stability and durability of MSC devices, Figure 4c displays the capacitance retention of MSC‐COF*_1.0‐0.6‐1.0_* and MSC‐COF*_3.9‐0.6‐3.9_* after CV charge–discharge cycles at 100 mV s^−1^. After 5000 cycles, the capacitance retention of MSC‐COF*_1.0‐0.6‐1.0_* and MSC‐COF*_3.9‐0.6‐3.9_* are ≈88.6% and 81.6% versus initial capacitance, respectively, indicating the superior durability of MSC‐COF. Moreover, no significant attenuation appears in their capacitance retention at different bending angles of 0°–180° (Figure 4d), verifying the good bending resistance. TEM (Figure S8, Supporting Information) characterization before and after long cycling shows no significant morphological changes, confirming its morphological stability. These features manifest that the COF*_x‐y‐x_* heterojunctions have high reliability even after repeated charge–discharge and arbitrary bending at large angles, which can guarantee the demand of future miniaturization and wearable electronic applications.^[^ ^35^ ^]^

The common self‐discharge phenomenon in supercapacitors is evaluated by collecting the potential signals after 10 charge/discharge cycles and standing for 2 h at 1 V. In Figure 4e, though most parallel experiment tests, the MSC‐COF*_1.0‐0.6‐1.0_* exhibits slow self‐discharge properties with its self‐discharge time up to 36 h, exceeding MSC‐COF*_3.9‐0.6‐3.9_*. This can be explained by the fact that the outer COF*_1.0_* with a higher H^+^ diffusion barrier of 0.75 eV can suppress the overflow of electrolyte H^+^ from the heterojunction, while the outer COF*_3.9_* with a large aperture (0.037 eV) has a strong tendency for H^+^ overflow.^[^ ^36^ ^]^ Upon comparison, the self‐discharge time of MSC‐COF*_1.0‐0.6‐1.0_* at a 100% discharge rate outlast that of some reported supercapacitors, including PVA‐10‐MSC,^[^ ^37^ ^]^ *n*‐Na*_x_* MnO_2_ versus *n*‐Na*_x_*_‐1_MnO_2_ (*n* = α, β, λ, γ, δ).^[^ ^38^ ^]^ In summary, the MSC‐COF*_1.0‐0.6‐1.0_* exhibits good electrochemical properties at the current level, such as excellent cycle stability, slow self‐discharge, high energy, and power density. A comparison of MSC‐COF*_1.0‐0.6‐1.0_* and COF*_3.9‐0.6‐3.9_* performance data is shown in Table S2 (Supporting Information**)**.

## 3 Conclusion

Sandwich‐type COF heterojunctions with various tailored stereoscopic apertures are prepared under *van der Waals* forces. Taking capacitance as the benchmark, the COF*_1.0‐0.6‐1.0_* heterojunction exhibits the optimal charge capacity: a volumetric specific capacitance of 598.6 F cm⁻^3^ and an energy density of 40.7 mWh cm^−3^ at a power density of 2095.2 mW cm^−3^. Theoretical calculations reveal that electrolyte H⁺ ions rapidly pass through the external COF*_1.0_* channels under the lamination effect derived from pore sizes and accumulate on the internal COF*_0.6_* interface, which is rich in active sites. This process not only achieves a high‐concentration electric double layer but also enhances the redox capacitance. The unique heterojunction structure effectively reduces the resistance to electron transfer, allowing electrons to conduct through in‐plane π‐electron clouds and facilitating *π–π* electron transitions across interfaces, thus achieving higher power output. Upon comparison with MSC‐COF*_3.9‐0.6‐3.9_*, the MSC‐COF*_1.0‐0.6‐1.0_* also possesses good self‐discharge property up to 36 h, where the outer COF*_1.0_* can also restrict H^+^ ions overflow causing self‐discharge properties, apart from aperture‐derived lamination effect on inner COF*_0.6_*, as well as charge accumulation layer. In addition, the MSC‐COF*_1.0‐0.6‐1.0_* owns excellent cycling and bending stability. These results indicate that COF heterojunctions have great potential and development prospects in the application of miniature or flexible/wearable devices.

## Conflict of Interest

The authors declare no conflict of interest.

## Supporting information



Supporting Information

## Data Availability

The data that support the findings of this study are available in the supplementary material of this article.
