# Supplementary material for: Covalent Organic Framework Nanofilm Heterojunctions: Lamination Effect and Suppressed Self‐Discharge in Flexible Micro‐Supercapacitors Energy Storage
Source: Small. 2025 Mar 27;22(12):2412642. doi: 10.1002/smll.202412642 (PMC12934377; doi:10.1002/smll.202412642)
Supplement: Supplementary file 1 — Supporting Information [file SMLL-22-2412642-s001.docx]

Supporting Information

**Covalent Organic Framework Nanofilm Heterojunctions: Lamination Effect and Suppressed Self-Discharge in Flexible Micro-Supercapacitors Energy Storage**

*Xiaoyang Xu, Tian Li, Ruijuan Zhang, Zihao Zhang, Wei Cao, Yue Wang, Yongqi Hu, Xinying Liu*, Shanlin Qiao**

X.Y. Xu, T. Li, R.J. Zhang, Z.H. Zhang, W. Cao, Y. Wang, Y.Q. Hu, S.L. Qiao

College of Chemistry and Pharmaceutical Engineering, Hebei University of Science and Technology, Shijiazhuang, 050018, China

Email: ccpeslqiao@hebust.edu.cn

R.J. Zhang, X.Y. Liu

Institute for Catalysis and Energy Solutions, University of South Africa, Private Bag X6, Florida, 1710, South Africa

Email: liux@unisa.ac.za

**Contents**

**1. Experimental Section**

1.1. Materials

1.2 Synthesis of COF Nanofilms and Heterojunctions

1.3 Non‑electrochemical Characterization

1.4 Electrochemical Characterization

1.5 Time-dependent density Functional Theory (TD-DFT) Calculations

**2. Results and Discussion**

**3. References1. Experimental Section**

**1.1 Materials**

The used monomers were served as follows: sodium dodecylbenzene sulfonate (SDBS, 98%) and 1,3,5-tris(4-aminophenyl) benzene (TAPB, 99.67%) from Tianjin Damao Chemical Reagent Factory, 1,3,6,8-Tetra-(p-aminophenyl)-pyrene (PyTA, 97%) and 2,3,6,7,10,11-hexaamino triphenyl hexahydro chloride (HATP, 95%) from Jilin Academy of Sciences-Yanshen Technology Co., Ltd., biphenyldicarboxaldehyde (BDC, 97%) and terephthalenaldehyde (PDA, 99%) from Shanghai Maclean's Biochemical Co., Ltd., 1,3,5-triaminobenzene trihydrochloride (BTA, 99.6%) from Shanghai Yien Chemical Technology Co., Ltd., 1,3,5-Benzenetricarboxaldehyde from Shanghai Haohong Biomedical Technology Co., Ltd. Chemicals. Other reagents such as CHCl_3_, acetic acid, poly (vinyl alcohol) (PVA, 97%), H_3_PO_4_ (85%) and hydrochloric acid chemicals, were obtained from Aladdin Industrial Corporation (Shanghai, China). All chemicals were used without further purification.

**1.2 Synthesis of COF Nanofilms and Heterojunctions**

The COF nanofilms were prepared by the gas-liquid interface synthesis technique, where SDBS (20 μL, 1 mg mL^-1^ in chloroform solvent) was dispersed into the deionized water (50 mL) in the crystallization dish to form static air-water interface. After the solvent evaporation for approximately 40 min, the monomer Ⅰ (HATP, 540 μL, 1 mg mL^-1^ in 0.12 M HCl solution) is injected into subaqueous aqueous phase for 1 h, followed by further adding monomer Ⅱ (TFB, 325 μL, 1 mg mL^-1^ in 0.12 M HCl solution). Under acetic acid (0.01 M, 2 mL) catalyst, the polymerization reaction of these two monomers was maintained at room temperature (~25 °C) for 7 days, to form the COF_HATP-TFB_ nanofilm. Considering the porous channel size matching optimization, the COF_HATP-TFB_ nanofilm was defined as COF*_0.6_* with the theoretical aperture of 0.6 nm by Materials Studio simulation.

Custom-made other COF*_n_* (*n*=1.0, 2.2, 2.7, 3.9) nanofilms were prepared on the basis of above interface synthesis method, except for corresponding monomers, such as BTCA (235 μL, 1 mg mL^-1^ in 0.12 M HCl solution) and TFB (165 μL, 1 mg mL^-1^ in 0.12 M HCl solution) for COF*_1.0_* nanofilms, PyTA (570 μL, 1 mg mL^-1^ in 0.12 M HCl solution) and PDA (270 μL, 1 mg mL^-1^ in 0.12 M HCl solution) for COF*_2.2_* nanofilms, PyTA (570 μL, 1 mg mL^-1^ in 0.12 M HCl solution) and BDC (420 μL, 1 mg mL^-1^ in 0.12 M HCl solution) for COF*_2.7_* nanofilms, TAPB (355 μL, 1 mg mL^-1^ in 0.12 M HCl solution) and BDC (315 μL, 1 mg mL^-1^ in 0.12 M HCI solution) for COF*_3.9_* nanofilms. A detailed summary of the data is shown in **Table S1**.

The COF*_x_* and COF*_y_* nanofilms were assembled into a sandwich-type COF*_x-y-x_* heterojunction by *van der Waals* forces. In this process, highly flat nanofilms are precisely transferred and ordered. Each layer of transferred COF film was first annealed and dried at 50 ℃. Then, the COF films were washed with water, ethanol, N, N-dimethylformamide (DMF), tetrahydrofuran, and acetone, which dissolved the COF monomers without destroying the COF framework, thereby removing the remaining monomers and impurities. After washing, the COF film was annealed and dried again. Finally, the solvent was completely evaporated to obtain a clean COF film [1, 2].

**Table S1.** Monomers and amounts used in the synthesis of COF films.

| COF | Monomer I and dosage | Monomer II and dosage |
| --- | --- | --- |
| COF_0.6_ | 2,3,6,7,10,11-hexaaminotriphenylene  (0.01 mmol) | 1,3,5-Benzenetricarboxaldehyde (0.02 mmol) |
| COF_1.0_ | 1,3,5-triaMine trihydrochloride  (0.01 mmol) | 1,3,5-Benzenetricarboxaldehyde (0.01 mmol) |
| COF_2.2_ | 4,4',4'',4'''-(pyrene-1,3,6,8-tetrayl)tetraaniline (0.01 mmol) | Terephthalaldehyde  (0.02 mmol) |
| COF_2.7_ | 4,4',4'',4'''-(pyrene-1,3,6,8-tetrayl)tetraaniline (0.01 mmol) | 4,4'-Biphenyldicarboxaldehyde  (0.02 mmol) |
| COF_3.9_ | 1,3,5-Tris(4-aminophenyl)benzene  (0.01 mmol) | 4,4'-Biphenyldicarboxaldehyde  (0.015 mmol) |

**1.3 Non‑electrochemical Characterization**

The COF nanofilms were firstly characterized by Fourier transform infrared spectroscopy (FT-IR) and X-ray photoelectron spectroscopy (XPS). Surface and phase structures of prepared COF nanofilms/heterojunctions were studied by optical microscopy (OM, 106 Leica ICC50 W), scanning (SEM, 107JEOL) and transmission electron microscopy (TEM, JEOL/JEM-2100). Further, the surface of COF*_0.6_*, COF*_1.0_* and COF*_1.0-0.6-1.0_* nanofilms/heterojunctions were also monitored by scanning electrochemical microscopy (SECM, VersaSCAN, Advance Measurement Technology, InC.) under the condition of K_3_Fe(CN)_6_/K_4_Fe(CN)_6_ electrolyte (0.1 mmol/L). The thickness of COF*_0.6_*, COF*_1.0_* nanofilms and COF*_1.0-0.6-1.0_* heterojunctions was measured by atomic force microscopy (AFM, Bruker Dimension Icon).

**1.4 Electrochemical Characterization**

The COF*_x-y-x_* heterojunctions were directly treated into interdigital electrodes by the mask plate technology, followed by injecting PVA/H_3_PO_4_ gel electrolyte into microelectrode channel, to obtain MSCs devices. The used PVA/H_3_PO_4_ gel electrolyte was prepared by mixing H_3_PO_4_ and PVA in deionized water at 90 ℃ for 1 h [3].

Energy storage properties of these MSC devices were studied on the VersaSTAT 3 electrochemical workstation through electrochemical measurements, especially cyclic voltammetry (CV). Important performance parameters of MSCs, such as areal (*C_A_*, mF cm^-2^) and volumetric (*C_V_*, F cm^-3^) specific capacitance, energy (*E*, Wh cm^-3^) and power density (*P*, W cm^-3^), were calculated based on equations (S1-4) [4], respectively.

$$\begin{aligned} C_{A}=\frac{\int_{V_{i}}^{V_{f}} I\left( V \right)dV}{2v\times A\times\left( V_{f}-V_{i} \right)}\#\left( S1 \right) \end{aligned}$$

$$\begin{aligned} C_{V}=\frac{\int_{V_{i}}^{V_{f}} I\left( V \right)dV}{2v\times V\times\left( V_{f}-V_{i} \right)}\#\left( S2 \right) \end{aligned}$$

where *ν* is the scan rate (V s^-1^), *V_𝑓_* and *V_𝑖_* are the integration voltage limits of *C–V* curve, 𝐼(V) is the voltametric current (A), *A* and *V* are the area (cm^2^) and volume (cm^3^) of the entire MSC devices.

$$\begin{aligned} E=\frac{1}{2}\times\frac{C_{V}\times\left( \Delta V \right)^{2}}{3600}\#\left( S3 \right) \end{aligned}$$

$$\begin{aligned} P=\frac{E\times3600}{\Delta t} \#\left( S4 \right) \end{aligned}$$

where Δ*V* and Δ*t* are the discharge voltage range (V) and discharge time (s), respectively.

Contribution rate of surface capacitance (*k_1_v*) and diffusion control (*k_2_v^1/2^*) of assembled COF heterojunctions are calculated by equation (S5) [5]:

$$\begin{aligned} i=k_{1}v+k_{2}v^{1/2}\#\left( S5 \right) \end{aligned}$$

Self-discharge duration properties of COF*_1.0-0.6-1.0_* and COF*_3.9-0.6-3.9_* were also measured in virtue of corresponding CR2032 symmetrical coin cell devices, on the LAND battery testing system.

The H^+^ transference number ($t_{H^{+}}$) of COF nanofilms was measured using the constant-voltage DC polarization method described by Evans *et al.* [6], as equation (S6).

$$\begin{aligned} t_{H^{+}}=\frac{I_{s}R_{e}^{s}\left( \Delta V-I_{0}R_{0} \right)}{I_{0}R_{e}^{0}\left( \Delta V-I_{s}R_{s} \right)}\#\left( S6 \right) \end{aligned}$$

where *I_0_* (*R_0_*) and *I_s_* (*R_S_*) are the initial and steady-state current (body resistance), *Re* is the electrolyte resistance.

**1.5 Time-dependent density Functional Theory (TD-DFT) Calculations**

The proton diffusion barrier of the COF structure was calculated in *Material Studio* software using the DMol3 calculation module to analyze the diffusion path and activation energy. Density functional theory (DFT) and the PBE function of generalized gradient approximation (GGA) were used to calculate the energy. This method has been widely used in the simulation of proton diffusion in metal oxides and porous materials. It also shows high reliability in barrier calculation [7]. In order to optimize the diffusion path, the Transition State Search (TS Search) method was used during the calculation. The complete LST/QST (Linear Synchronous Transit/Quadratic Synchronous Transit) protocol was used to locate the transition state structure. The core electrons were treated with DFT Semi-core Pseudopotential, and a dual-numerical (DN) basis set was selected to improve the calculation accuracy. A convergence threshold of 1.0×10^-4^ Ha was set for self-consistent field (SCF) calculation, and the maximum number of iteration steps was 50 to ensure the stability and convergence of the calculation. At the same time, *Multipolar Expansion* was introduced and calculated to the level of sixteenth pole moment. Optimized electrostatic potential calculation [8]. In addition, in order to improve the computational efficiency and convergence, the density hybrid method was used for charge optimization, and the direct repeated iterative solution (DIIS) algorithm was used to accelerate the convergence, and the DIIS subspace size was set to 6. The electron temperature broadening (Smearing) technique was used to calculate the orbital occupancy, and the broadening parameter was set to 0.06 Ha to reduce the discontinuity of orbital filling. The main output of the calculation includes diffusion path, transition state structure and diffusion barrier. The results show that the pore size in the COF structure has a significant effect on proton diffusion, and the TS Search method can effectively identify the lowest energy diffusion path in porous materials. [9].

**2. Results and Discussion**

**Fig. S1** (a-e) The chemical structure in AA stacking model. (f) FT-IR spectra of COFs; The XPS full spectra (g), XPS C 1*s* spectra (h), and XPS N 1*s* spectra (i) of COFs.

**Fig. S2** (a-e) FT-IR spectra of bulk COF*_n_* (*n*=0.6, 1.0, 2.2, 2.7, 3.9).

**Fig. S3** (a-e) P-XRD patterns of bulk COF*_n_* (*n*=0.6, 1.0, 2.2, 2.7, 3.9)

**Fig. S4** (a-c) The force-distance curves of COF*_0.6_*, COF*_1.0_* and COF*_1.0-0.6-1.0_*.

**Fig. S5** *C–V* curves of MSC-COF*_3.9-1.0-3.9_* (a), MSC-COF*_3.9-2.2-3.9_* (b), MSC-COF*_3.9-2.7-3.9_* (c), MSC-COF*_3.9-3.9-3.9_* (d).

**Fig. S6** *C–V* curves of MSC-COF*_0.6-0.6-0.6_* (a), MSC-COF*_2.2-0.6-2.2_* (b), MSC-COF*_2.7-0.6-2.7_* (c), MSC-COF*_3.9-0.6-3.9_* (d).

**Fig. S7** (a) *C_A_* of MSC-COF*_1.0-0.6-1.0_,* COF*_1.0_* and COF*_0.6_*, (b) EIS plots before and after timing current.

**Fig. S8** TEM images before and after long cycling of COF*_1.0-0.6-1.0_* heterojunction.

**Table S2.** Comparison of MSC-COF heterojunction performance data.

| MSC-COF heterojunction | *C_A_*  (mF cm^-2^) | $t_{H^{+}}$ | *C_dl_*  (mF cm^-2^) | Cycling stability (%) | Self-discharge (h) |
| --- | --- | --- | --- | --- | --- |
| MSC-COF*_1.0-0.6-1.0_* | 0.2358 | 0.81 | 0.0273 | 0.886 | 36.6 |
| MSC-COF*_3.9-0.6-3.9_* | 0.1501 | 0.62 | 0.0189 | 0.816 | 7.2 |

**C_A_* values at 100 mV s^-1^

**3.** **References**

1. K. Kamal, M. A. Bustam, M. Ismail, D. Grekov, A. Mohd Shariff, P. Pré, Materials **2020**, 13, 2741.

2. M. S. Lohse, J. M. Rotter, J. T. Margraf, V. Werner, M. Becker, S. Herbert, P. Knochel, T. Clark, T. Bein, D. D. Medina, CrystEngComm **2016**, 18, 4295.

3. Zhang, Z.; Xu, X.; Xing, X.; Tang, X.; Zhang, X.; Chen, J.; Xu, Y.; Jiang, H., *Appl. Surf. Sci.* **2025,** *680*, 161327.

4. Jiang, K.; Baburin, I. A.; Han, P.; Yang, C.; Fu, X.; Yao, Y.; Li, J.; Cánovas, E.; Seifert, G.; Chen, J.; Bonn, M.; Feng, X.; Zhuang, X., *Adv. Funct. Mater.* **2019,** *30* (7), 1908243.

5. Kavan, L.; Grätzel, M.; Rathouský, J.; Zukalb, A., *J. Electrochem. Soc.* **2019,** *143* (2), 394–400.

6. Evans, J.; Vincent, C. A.; Bruce, P. G., *Polymer* **1987,** *28* (13), 2324-2328.

7. X. Pan, W. Yang, Y. Xie, Y. Chen, H. Guo, J. Phys. Chem. C **2021**, 125, 3804.

8. M. A. Gomez, M. A. Griffin, S. Jindal, K. D. Rule, V. R. Cooper, J. Chem. Phys. **2005**, 123, 094703.

9. T. Bučko, L. Benco, J. Hafner, J. G. Ángyán, J. Catal. **2011**, 279, 220.
